# Analysis of regional characteristics in mortality trends of three chronic infectious diseases among the elderly in China, 2004–2021

**DOI:** 10.1186/s40249-025-01345-1

**Published:** 2025-07-17

**Authors:** Yi-ran Xiao, Xiang Ren, Meng-di Zhang, He Zhu, Xin Wang, Wen-shan Sun, Xiao-min Guo, Fei Huang, Jian-jun Liu, Hong-yan Yao, Qi-qi Wang, Wen-jing Zheng

**Affiliations:** 1 Chinese Center for Disease Control and Prevention, Beijing, China 102206; 2 Peking University, Beijing, 100871 China; 3 Guang’anmen Hospital, China Academy of Chinese Medical Sciences, Beijing, 100053 China

**Keywords:** Three chronic infectious diseases, HIV/AIDS, Hepatitis B, Tuberculosis, Mortality trends, Elderly, Joinpoint regression, Regional characteristics, China

## Abstract

**Background:**

HIV/AIDS, hepatitis B, and tuberculosis (TB) are chronic infectious diseases prioritized by the United Nations Sustainable Development Goals (SDGs) and China’s “Healthy China Initiative (2019–2030),” posing persistent challenges to global and Chinese public health systems. This study analyzed mortality trends and regional/urban-rural disparities of these three diseases among Chinese elderly individuals aged 60 years and older from 2004 to 2021 to identify priority areas for targeted prevention strategies.

**Methods:**

Data were sourced from the “China Cause-of-Death Surveillance Dataset (2004–2021)” published by the Chinese Center for Disease Control and Prevention. The study population comprised Chinese elderly individuals aged 60 years and older from 2004 to 2021. Joinpoint 4.9.0.1 software was used for joinpoint regression analysis to characterize dynamic mortality trends, calculating average annual percentage change (AAPC), annual percentage change (APC), and 95% confidence intervals (*CI*s).

**Results:**

From 2004 to 2021, 100,934 deaths from the three chronic infectious diseases occurred among Chinese elderly. The overall age-standardized mortality rate (ASMR) decreased from 51.00 to 13.37 per 100,000 (AAPC: − 7.54%, 95% *CI:* − 9.38%, − 5.67%). Notably, the ASMR for HIV/AIDS increased from 0.11 to 0.85 per 100,000 population, while the ASMR for hepatitis B declined from 17.96 to 6.84 per 100,000 population and TB declined from 32.92 to 5.68 per 100,000 population. The three chronic infectious diseases collectively demonstrated the most significant ASMR reduction in central China (AAPC: − 7.85%, 95% *CI:* − 12.11%, − 3.39%), followed by eastern China (− 7.57%, 95% *CI:* − 9.02%, − 6.09%) and western China (− 7.10%, 95% *CI:* − 9.52%, − 4.62%). Rural areas experienced substantially steeper ASMR declines compared to urban areas (AAPC: − 7.81%, 95% *CI:* − 9.40%, − 6.18% vs. AAPC: − 6.05%, 95% *CI:* − 8.20%, − 3.85%).

**Conclusion:**

China achieved a continuous decline in the overall ASMR for the three chronic infectious diseases among elderly populations during 2004–2021, suggesting initial success in prevention efforts. However, the rising ASMR for HIV/AIDS necessitates disease-specific strategies. Notable regional disparities persist: hepatitis B and TB remain concentrated in western/rural areas, while the ASMR for HIV/AIDS is higher in urban regions. Future efforts should tailor resource allocation to local contexts to enhance intervention outcomes and protect elderly health.

## Background

Human immunodeficiency virus/acquired immunodeficiency syndrome (HIV/AIDS), hepatitis B, and tuberculosis (TB) (hereinafter referred to as the “three chronic infectious diseases”) are significant public health challenges explicitly targeted for elimination in the United Nations Sustainable Development Goals (SDGs). Their prevention and control outcomes have a crucial impact on global public health security [1]. In *Healthy China Action (2019*–*2030)*, these three chronic infectious diseases are listed as priority control targets due to their shared characteristics of high infectivity, prolonged disease course, and the need for long-term management, which impose sustained pressure on public health systems [2].

As the global process of population aging accelerates, the elderly population is gradually emerging as the primary susceptible group for these three chronic infectious diseases [3–5]. According to Global Burden of Disease (GBD) data, in 2021, deaths caused by these three diseases among people aged 60 and above in China reached 36,015 cases, accounting for 49.88% of the total national deaths from these diseases. Meanwhile, the disability-adjusted life years (DALYs) lost amounted to 929,269 person-years [6]. Although China’s infectious disease prevention and control system has continuously improved, challenges such as complex transmission routes, diversified epidemic patterns, and limited control resources for major chronic infectious diseases persist, particularly among the elderly, who bear a significant disease burden and experience health inequities [7, 8]. A thorough analysis of the epidemic trends and evolution patterns of the three chronic infectious diseases among elderly populations in different regions of China holds crucial strategic significance for precisely optimizing the country's infectious disease prevention and control strategies and enhancing the response effectiveness of the public health system.

Existing research has predominantly focused on single diseases or localized regions, lacking systematic analysis of the overall spatiotemporal evolution patterns of the three chronic infectious diseases. This has somewhat constrained decision-making support for the precise allocation of prevention and control resources. In light of this, this study breaks through the traditional single-disease research framework and innovatively integrates HIV/AIDS, hepatitis B, and TB as a composite research subject termed the “three chronic infectious diseases”. This study aims to analyze the mortality trends and assess regional (eastern, central, and western) and urban-rural disparities in the prevalence of three chronic infectious diseases among China’s elderly population from 2004 to 2021.

## Methods

### Investigated subject

This study collected mortality surveillance data from 2004 to 2021 for three chronic infectious diseases—TB, hepatitis B, and AIDS—among the elderly population aged 60 years and older, as documented in the *China Cause of Death Surveillance Dataset (2004–2021)* compiled by the Chinese Center for Disease Control and Prevention. Statistical analyses were conducted to stratify the dataset by gender (male/female), age group (60–64, 65–69, 70–74, 75–79, 80–84, and ≥ 85 years), residence type (urban/rural), and geographic region (eastern, central, and western China).

### Data source

The mortality data for the three chronic infectious diseases among the elderly aged 60 years and older between 2004 and 2021 were obtained from the *China Cause of Death Surveillance Dataset (2004–2021)* [9], compiled by the Chinese Center for Disease Control and Prevention (China CDC). The three chronic infectious diseases included in this study were TB, hepatitis B, and AIDS, with corresponding ICD-10 codes A15–A19, B90, and B16–B19 (excluding B17.1 and B18.2), and B20–B24, respectively. The study covered 31 provinces, autonomous regions, and municipalities in the Chinese mainland (not included Hong Kong, Macau, and Taiwan). For regional classification, all prefecture-level cities in Chinese mainland were divided into three regions: eastern, central, and western. The eastern region included Beijing, Tianjin, Hebei, Liaoning, Shanghai, Jiangsu, Zhejiang, Fujian, Shandong, Guangdong, and Hainan. The central region comprised Shanxi, Jilin, Heilongjiang, Anhui, Jiangxi, Henan, Hubei, and Hunan. The western region included Inner Mongolia, Guangxi, Chongqing, Sichuan, Guizhou, Yunnan, Xizang, Shaanxi, Gansu, Qinghai, Ningxia, and Xinjiang. Urban and rural classifications defined all counties (including county-level cities) as rural and all districts as urban.

Resident population data for China were sourced from the Comprehensive Disease Prevention and Control Information System of the China CDC. Standard population data were obtained from the "Tabulation on the 2010 Population Census of China," compiled by the Population Census Office of the State Council and the Department of Population and Employment Statistics of the National Bureau of Statistics [10].

The *China Cause of Death Surveillance Dataset (2004–2021)* integrated population and mortality data from the national Disease Surveillance Points (DSPs) system from 2004 to 2021. Erroneous data were corrected, and implausible data were excluded to ensure data authenticity [11]. To maintain high quality and reliability, the dataset underwent data quality assessment and cleaning before analysis and result compilation.

### Statistical indicators

The mortality rates of the three chronic infectious diseases among the elderly aged 60 years and older from 2004 to 2021 were calculated by urban/rural residence and region. To eliminate the influence of age distribution differences across years when analyzing mortality trends, age standardization was performed using the age composition from the 2010 Sixth National Population Census [10]. The age-standardized rate (ASR) was calculated as $ASR$ = $\sum ({N}_{i}{P}_{i})$/$N$, where *N*_*i*_ is the population of the *i*-th age group in the standard population, *P*_*i*_ is the crude mortality rate of the *i*-th age group, and *N* is the total standard population.

### Statistical analysis

This study employed the Joinpoint regression model to describe the temporal trends in mortality rates of the three chronic infectious diseases among the elderly aged 60 years and older in China from 2004 to 2021. The Joinpoint regression model [12] is a collection of linear statistical models, where "joinpoints" refer to the points connecting all models. This model effectively identifies turning points in mortality trends and calculates the average percent change for each segment, providing a more accurate reflection of dynamic changes in mortality rates compared to traditional linear trend analysis [13, 14]. The Joinpoint regression model includes linear (y = xb) and log-linear (ln y = xb) models. The linear model is used for normally distributed data, while the log-linear model is suitable for exponentially or Poisson-distributed data. Given that population-based disease mortality trends typically follow a log-linear distribution [15], this study adopted the log-linear model.

The grid search method (GSM) was used for joinpoint analysis and parameter estimation, at the same time, the Monte Carlo permutation test was applied to select the optimal model and identify statistically significant trend change points. The APC and AAPC from 2004 to 2021, along with their 95% confidence intervals (*CI)*, were calculated. If the 95% *CI* did not include zero, the APC and AAPC were considered statistically significant, with positive or negative values indicating "increasing" or “decreasing” trends, respectively. The magnitude of the absolute value reflected the extent of change. If no statistical significance was observed, the trend was labeled as "stable." If no joinpoints were identified, APC = AAPC, indicating a monotonic increasing or decreasing trend. A two-sided test was used, with α = 0.05 and *P* < 0.05 considered statistically significant [16].

## Results

### Mortality from three chronic infectious diseases among the elderly in China, 2004–2021

From 2004 to 2021, a total of 100,934 deaths from three chronic infectious diseases were recorded among the Chinese population aged 60 and above, and among these, males and females accounted for 67.79% (68,423) and 32.21% (32,511), respectively. The distribution by region was 28.02% (28,284) in the eastern region, 32.13% (32,430) in the central region, and 39.85% (40,220) in the western region. Urban and rural areas accounted for 28.11% (28,373) and 71.89% (72,561) respectively. The majority of deaths occurred in the age groups of 65–69 and 70–74 years, totaling 40,041 cases, which constituted 39.67% of all elderly deaths (Table 1).

**Table 1:** Basic situation of three chronic infectious diseases among the elderly aged 60 and above in China, 2004–2021 (*n* = 100,934)

Year	*n*	Gender (%)		Age (%)						District (%)			Urban and Rural (%)	
	Overall	Male	Female	60–64	65–69	70–74	75–79	80–84	85–89	Eastern	Central	Western	Urban	Rural
2004	4102	65.50	34.50	18.43	22.77	22.62	17.43	11.92	6.83	25.11	33.08	41.81	23.11	76.89
2005	4173	65.61	34.39	18.33	20.78	22.74	18.93	11.38	7.84	24.66	32.71	42.63	22.26	77.74
2006	2824	68.24	31.76	19.33	21.42	23.97	18.20	11.33	5.74	31.09	32.33	36.58	22.59	77.41
2007	2571	67.76	32.24	19.49	19.88	23.14	17.35	13.22	6.92	29.95	32.24	37.81	27.46	72.54
2008	2695	69.54	30.46	18.48	20.59	22.52	20.26	11.65	6.49	30.61	32.80	36.59	26.83	73.17
2009	2496	70.03	29.97	17.11	19.43	22.56	20.11	13.38	7.41	30.05	31.25	38.70	27.72	72.28
2010	2362	67.82	32.18	17.82	19.90	20.36	18.97	14.56	8.38	29.47	28.58	41.96	32.56	67.44
2011	2270	68.15	31.85	20.31	19.30	20.22	19.74	12.82	7.62	25.81	30.00	44.19	29.43	70.57
2012	2290	67.60	32.40	19.91	18.08	20.44	21.27	12.79	7.51	26.29	28.91	44.80	28.78	71.22
2013	7237	69.03	30.97	20.59	19.52	18.79	18.89	13.21	9.00	29.20	31.01	39.80	26.99	73.01
2014	8166	68.66	31.34	19.69	19.31	19.28	19.29	14.25	8.18	28.04	35.81	36.15	27.10	72.90
2015	8589	67.07	32.93	19.56	19.25	18.19	19.05	14.25	9.71	28.26	33.50	38.25	28.37	71.63
2016	8980	67.33	32.67	20.13	19.06	18.06	18.39	13.95	10.40	27.85	31.87	40.28	28.64	71.36
2017	8927	67.94	32.06	19.95	20.07	17.79	18.07	13.77	10.35	29.47	30.98	39.54	28.49	71.51
2018	8642	67.43	32.57	19.61	19.76	18.31	17.47	13.62	11.22	28.86	31.22	39.92	29.01	70.99
2019	8776	68.22	31.78	17.34	20.51	18.87	17.79	13.87	11.62	27.77	32.22	40.01	30.47	69.53
2020	8286	67.92	32.08	16.50	21.36	19.32	17.45	13.75	11.62	27.01	32.15	40.84	30.53	69.47
2021	7548	67.47	32.53	15.49	20.84	19.78	17.12	14.53	12.24	26.32	32.07	41.60	29.31	70.69
Overall	100,934	67.79	32.21	18.78	20.09	19.58	18.38	13.53	9.65	28.02	32.13	39.85	28.11	71.89

**Fig. 1:**
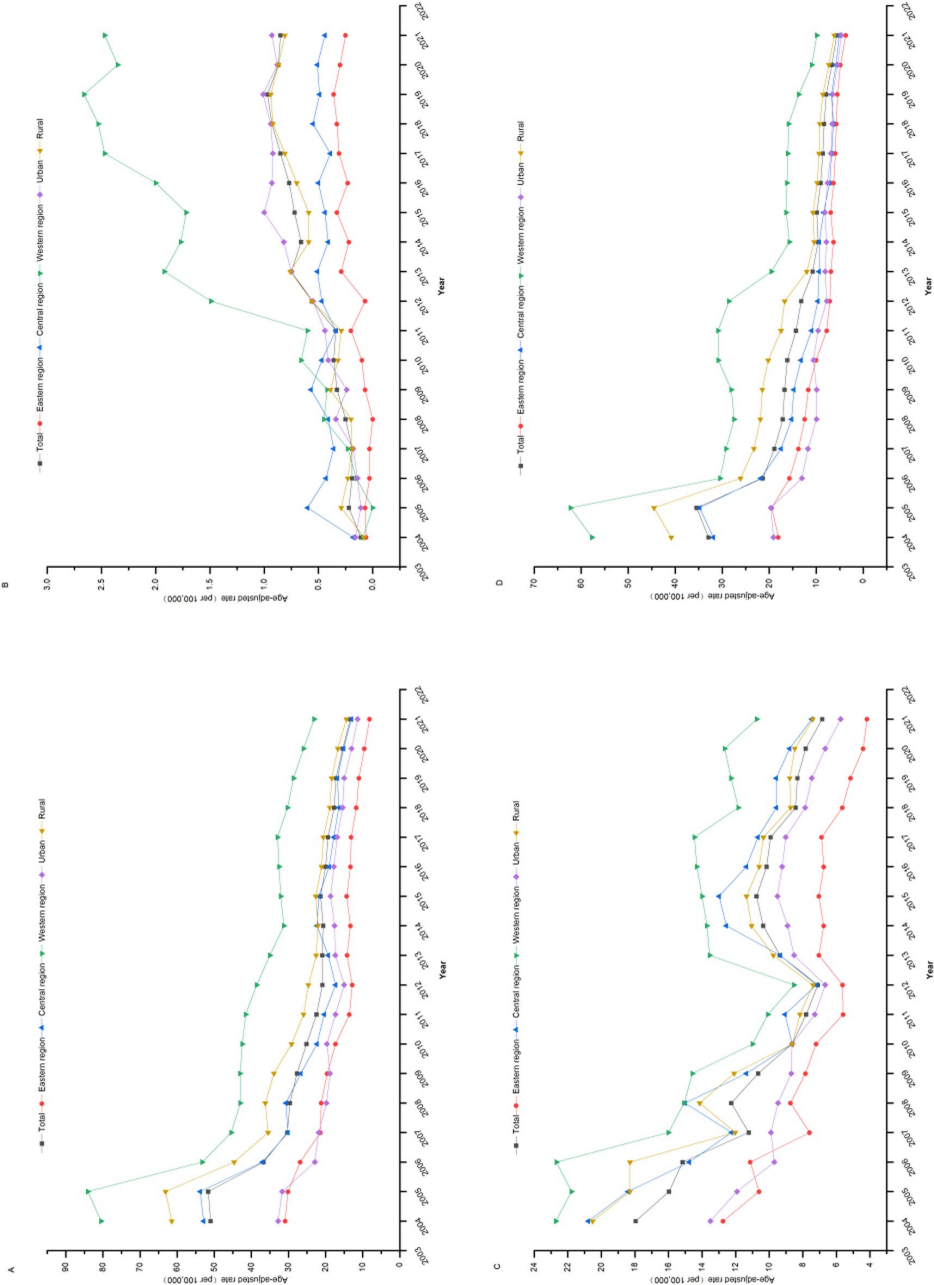
Temporal trends in standardized mortality rates of HIV/AIDS, hepatitis B and tuberculosis (TB) in China during 2004–2021. **A**: Three chronic infectious diseases; **B**: HIV/AIDS; **C**: hepatitis B; **D**. TB

From 2004 to 2021, the ASMR for the three chronic infectious diseases among the elderly in China declined from 51.00 per 100,000 to 13.37 per 100,000. Specifically, the ASMR for HIV/AIDS increased from 0.11 per 100,000 to 0.85 per 100,000, while hepatitis B decreased from 17.96 per 100,000 to 6.84 per 100,000, and TB declined from 32.92 per 100,000 to 5.68 per 100,000. Regional stratification revealed that, from 2004 to 2021, the overall ASMR for the three chronic infectious diseases was highest in the western region, followed by the central and eastern regions, respectively. For HIV/AIDS, the western region had the highest ASMR from 2010 to 2021, followed by the central and eastern regions. Similarly, hepatitis B and TB exhibited higher ASMRs in the western region compared to the central and eastern regions from 2004 to 2021. Urban-rural stratification showed that, from 2004 to 2021, the ASMRs for the three chronic infectious diseases, hepatitis B and TB were higher in rural areas than in urban areas. However, for HIV/AIDS, the ASMR was higher in urban areas than in rural areas from 2014 to 2021 (Fig. 1).

### Trend analysis on mortality of three chronic infectious diseases among Chinese elderly in diverse regions, 2004–2021

#### Joinpoint regression analysis of mortality trends for three chronic infectious diseases between urban and rural areas in China

From 2004 to 2021, the ASMR of three chronic infectious diseases among the elderly population in China exhibited an overall declining trend, with an AAPC of − 7.54% (95% *CI:* − 9.38%, − 5.67%). This downward trend was observed in all regions and across both urban and rural areas, with rural areas showing a more pronounced decline than urban areas. Specifically, the AAPC for rural areas was − 7.81% (95% *CI:* − 9.40%, − 6.18%), while the AAPC for urban areas was − 6.05% (95% *CI:* − 8.20%, − 3.85%).

Through segmented Joinpoint regression analysis, two change points were identified in the ASMR trend for the three chronic infectious diseases among the elderly in China from 2004 to 2021. Both periods, 2004–2010 and 2017–2021 showed declining trends, with APCs of − 12.94% (95% *CI:* − 16.39%, − 9.35%) and − 8.42% (95% *CI:* − 12.65%, − 3.98%), respectively. Stratified by urban-rural division, urban areas had two change points, with declining trends in both 2004-2008 and 2017-2021, exhibiting APCs of − 13.86% (95% *CI:* − 20.85%, − 6.25%) and − 9.35% (95% *CI:* − 13.72%, − 4.75%), respectively. Rural areas had one change point, with declining trends in both 2004–2010 and 2010–2021, showing APCs of − 12.76% (95% *CI:* − 16.80%, − 8.52%) and − 4.98% (95% *CI:* − 6.37%, − 3.58%) (Table 2).

**Table 2:** Results of Joinpoint regression analysis on mortality rates of three chronic infectious diseases across different urban–rural and eastern-central-western regions in China, 2004–2021

Type	Year	ASMR (per 100,000)	APC (95% *CI*, %)	AAPC (95% *CI*, %)
Overall	2004–2010	51.00–25.15	− 12.94*(− 16.39, − 9.35)	−7.54*(− 9.38, − 5.67)
	2010–2017	25.15–19.35	− 2.12(− 5.37, 1.25)	
	2017–2021	19.35–13.37	− 8.42*(−12.65, − 3.98)	
District				
Eastern	2004–2012	30.88–12.81	− 10.02*(− 12.02, − 7.96)	− 7.57*(− 9.02, − 6.09)
	2012–2017	12.81–13.11	− 0.47(− 4.21, 3.42)	
	2017–2021	13.11–8.15	− 11.08*(− 14.49, − 7.53)	
Central	2004–2011	52.85–20.36	− 14.68*(− 17.99, − 11.25)	− 7.85*(− 12.11, − 3.39)
	2011–2014	20.36–22.32	6.66(− 19.81, 41.87)	
	2014–2021	22.32–13.03	− 6.52*(− 8.67, − 4.32)	
Western	2004–2007	80.51–45.42	− 19.07*(− 30.62, − 5.61)	− 7.10*(− 9.52, − 4.62)
	2007–2021	45.42–23.10	− 4.32*(− 5.51, − 3.11)	
Urban and Rural				
Urban	2004–2008	32.77–19.78	− 13.86*(−20.85, − 6.25)	− 6.05*(− 8.20, − 3.85)
	2008–2017	19.78–16.85	− 0.79(−3.14, 1.62)	
	2017–2021	16.85–11.38	− 9.35*(−13.72, − 4.75)	
Rural	2004–2010	61.45–29.16	− 12.76*(− 16.80, − 8.52)	− 7.81*(− 9.40, − 6.18)
	2010–2021	29.16–14.40	− 4.98*(− 6.37, − 3.58)	

^*^Indicates that the APC or the AAPC is significantly different from zero at the alpha = 0.05 level. Abbreviations: *ASMR* Age-standardized mortality rate, *APC* Annual percent change, *AAPC* Average annual percentage change, *CI* Confidence intervals

#### Joinpoint regression analysis of mortality trends for three chronic infectious diseases across eastern, central, and western regions of China, 2004–2021

From 2004 to 2021, the ASMR for the three chronic infectious diseases among the elderly in China displayed declining trends across the eastern, central, and western regions. The most significant decline occurred in the central region, with an AAPC of –7.85% (95% *CI*: –12.11%, –3.39%), followed by the eastern region with an AAPC of − 7.57% (95% *CI:* − 9.02%, − 6.09%). The smallest decline was observed in the western region, with an AAPC of − 7.10% (95% *CI:* − 9.52%, − 4.62%).

Segmented joinpoint regression analysis revealed regional variations. The eastern region had two change points, with declining trends in both 2004–2012 and 2017–2021, showing APCs of –10.02% (95% *CI:* − 12.02%, − 7.96%) and − 11.08% (95% *CI:* − 14.49%, − 7.53%), respectively. The central region also had two change points, with declining trends in 2004–2011 and 2014–2021, exhibiting APCs of − 14.68% (95% *CI:* − 17.99%, − 11.25%) and –6.52% (95% *CI:* − 8.67%, − 4.32%), respectively. The western region had one change point, with declining trends in both 2004–2007 and 2007–2021, displaying APCs of − 19.07% (95% *CI:* − 30.62%, − 5.61%) and − 4.32% (95% *CI:* − 5.51%, − 3.11%) (Table 2).

## Discussion

This study systematically analyzed the mortality trends and regional distribution characteristics of three chronic infectious diseases—HIV/AIDS, hepatitis B, and TB—among the elderly population aged 60 and above in China from 2004 to 2021, based on data from the National Disease Surveillance System. The findings revealed an overall declining trend in the ASMRs of these three chronic infectious diseases, though significant regional heterogeneity and urban-rural disparities were observed. These results provide crucial scientific evidence for optimizing coordinated prevention and control strategies for multiple chronic infectious diseases among the elderly in China.

This study demonstrated that the ASMRs of the three chronic infectious diseases among the elderly in China exhibited an overall downward trend from 2004 to 2021. The most substantial annual decline in ASMRs occurred between 2004 and 2010, which coincided with key milestones in China’s public health system reforms. In 2004, revisions to the *Law on the Prevention and Treatment of Infectious Diseases* enhanced the efficiency of the infectious disease reporting system, thereby improving case surveillance and emergency response capabilities [17]. At the disease-specific level, the ASMR of HIV/AIDS exhibited an upward trend, which is consistent with findings from other studies [18]. From 2004 to 2021, China implemented a series of policies explicitly aimed at “expanding surveillance network coverage and improving the efficiency of case detection” [19]. However, due to the lack of definitive curative treatments for HIV, limited drug options, and growing antiviral resistance [20], HIV/AID-related mortality has shown an upward trend in recent years. The results of this study reveal that the ASMRs of hepatitis B and TB both demonstrated a downward trend, which aligns with findings from other research [21, 22]. The declining mortality rates of hepatitis B and TB reflect the effectiveness of China’s prevention and control programs, as well as the sustained improvements in socioeconomic and healthcare conditions that underpin these efforts [23–25].

Regional analysis results indicate that from 2004 to 2021, the ASMRs of the three chronic infectious diseases among the elderly in western China were higher than those in eastern and central regions, with an annual average decline lower than that in eastern and central regions. Specifically, the ASMRs of HIV/AIDS from 2010 to 2021, and hepatitis B and TB from 2004 to 2021, followed the pattern: western > central > eastern regions. This aligns with the "Hu Line" phenomenon in China’s healthcare resource allocation [26]. Studies indicate that in 2012, the number of patients with severe diseases treated in western China was significantly lower than in eastern and central regions [27]. In 2019, the capacity for primary prevention services for vaccine-preventable diseases (VPDs) in western China lagged behind eastern and central regions, with lower service coverage and accountability [28]. Furthermore, scholars such as Wan [29] have highlighted issues such as low assistance levels, insufficient funding, suboptimal implementation, weak assistance intensity, and inadequate coordination with other systems in the "Critical Illness Medical Assistance" policy for western China. This structural imbalance directly undermines the effectiveness of disease control measures. Meanwhile, delayed healthcare access due to geographic barriers, cultural and cognitive differences in multi-ethnic communities, and disease transmission risks associated with labor migration [30–32] collectively pose special challenges for disease prevention and control in western China. These findings suggest that the state should increase investment and support for western China, enhance primary healthcare capacity, and strengthen prevention and control efforts for the three chronic infectious diseases. Western regions should learn from the experiences of eastern and central regions and promote effective prevention and control measures.

Urban–rural analysis results indicate that from 2004 to 2021, the annual average decline in the ASMRs of the three chronic infectious diseases among the elderly in rural areas was greater than that in urban areas. This phenomenon may be attributed to various factors, including the continuous enhancement of health awareness, gradual improvement in lifestyles, and optimization of sanitary conditions in rural areas [33, 34]. However, it is noteworthy that the ASMRs in rural areas consistently remained higher than those in urban areas, with a significant gap in the composition of deaths between urban and rural areas, highlighting the structural deficiencies in China’s dual healthcare system. This disparity may be due to factors such as the location of initial medical consultations, uneven distribution of medical resources, and the presence of empty-nest elderly in rural areas [35–38]. Additionally, the current lack of interoperability in electronic health records within county-level healthcare networks may contribute to this issue [39]. At the disease-specific level, the ASMR of HIV/AIDS was higher in urban areas than in rural areas. This may be attributed to urban residents often having better access to behavioral determinants compared to rural populations [40]. Furthermore, the acceleration of urbanization has led to the concentration of a large number of sexually active and self-protection-conscious mobile populations in urban areas, especially high-risk groups such as men who have sex with men (MSM), increasing the risk of HIV transmission and resulting in relatively higher HIV/AIDS mortality rates in urban areas [41–43]. It is recommended to strengthen HIV/AIDS health knowledge dissemination among urban populations, focusing on mobile populations and high-risk groups.

A key strength of this study is its nationwide data coverage and regional-level analysis. Unlike prior studies [44], this research utilized data from the DSP of the China CDC, rather than the National Notifiable Disease Reporting System (NNDRS) [45]. The NNDRS captures mortality among treated patients, defined as deaths from any cause during treatment, whereas the DSP records deaths attributed to chronic infectious diseases by physicians. However, several limitations should be noted. First, as the study relied on registry data, incomplete information may exist. While the DSP is the only national mortality surveillance system covering all causes of death in China, actual mortality figures for the three chronic infectious diseases may exceed reported values. Second, although joinpoint regression models effectively describe dynamic trends in chronic infectious disease mortality [46], they cannot elucidate underlying causes or establish causal relationships [47]. Third, regional and urban–rural comparisons may be confounded by unmeasured factors, such as economic status and healthcare service availability, which were not analyzed in this study. Future research should incorporate these determinants better to understand their impact on disease epidemiology and control.

## Conclusions

This study focuses on the prevention and control effectiveness and challenges of three chronic infectious diseases (HIV/AIDS, hepatitis B, and TB) among the elderly population in China. The research findings indicate that China has achieved remarkable progress in the overall prevention and control of the three chronic infectious diseases among the elderly. However, significant disparities in ASMRs exist between the eastern, central, and western regions, as well as between urban and rural areas. Specifically, the ASMR of HIV/AIDS among the elderly is on the rise, with the epidemic mainly concentrated in urban areas. Although the ASMRs of hepatitis B and TB have shown a downward trend, the epidemic situation remains severe in the western regions and rural areas. These discoveries underscore the importance of targeted and precise prevention and control measures for the elderly population, providing crucial evidence for further optimizing prevention and control strategies.

### Recommendations

To effectively enhance the prevention and control level of the three chronic infectious diseases among the elderly population, this study proposes the establishment of a tiered and precise prevention and control system, as follows:

- For urban regions with a high prevalence of HIV/AIDS among the elderly: Strengthen multi-sectoral collaborative governance. The health department should take the lead and collaborate closely with departments such as civil affairs, education, and community organizations. The aim is to integrate resources and share information. The civil affairs department should focus on the living conditions of vulnerable elderly groups to facilitate targeted assistance. The education department should carry out awareness campaigns on HIV/AIDS prevention and control among the elderly. Community organizations should conduct regular health monitoring and interventions to prevent poverty caused by illness, thereby improving the overall effectiveness of HIV/AIDS prevention and control in urban areas for the elderly.
- For western and rural regions with a high risk of hepatitis B and TB among the elderly: Implement a "province-to-county" collaboration model. Provincial medical institutions should leverage their technological advantages to enhance the screening and diagnostic capabilities of county-level medical institutions for elderly patients. This can be achieved through technical guidance, personnel training, and telemedicine. Meanwhile, strengthen follow-up management at the grassroots level to improve treatment adherence.

## Data Availability

The datasets analyzed during this study are available from the corresponding author on reasonable request.

## Abbreviations

AAPC: Average annual percentage change
APC: Annual percent change
ASMR: Age-standardized mortality rate
ASR: The age-standardized rate
*CI*: Confidence intervals
DALYs: Disability-adjusted life years
DSPs: Disease surveillance points
GBD: Global burden of disease
GSM: The grid search method
HIV/AIDS: Human immunodeficiency virus/acquired immunodeficiency syndrome
ICD: International classification of diseases
MSM: Men who have sex with men
NNDRS: National notifiable disease reporting system
SDGs: The United Nations sustainable development goals
TB: Tuberculosis
VPDs: Vaccine preventable diseases
